# Breast cancer stromal fibroblasts promote the generation of CD44^+^CD24^- ^cells through SDF-1/CXCR4 interaction

**DOI:** 10.1186/1756-9966-29-80

**Published:** 2010-06-22

**Authors:** Mingzhu Huang, Yuqing Li, Huanle Zhang, Feifei Nan

**Affiliations:** 1 Department of Oncology, Xinhua Hospital, Shanghai Jiao Tong University School of Medicine, Shanghai 200092, China; 2 College of Chemistry, Nanjing University, Nanjing, China; 3 Shanghai Jiao Tong University School of Medicine, Shanghai, China

## Abstract

**Background:**

Breast cancer stem cells (BCSCs) have been recently identified in breast carcinoma as CD44^+^CD24^- ^cells, which exclusively retain tumorigenic activity and display stem cell-like properties. Using a mammosphere culture technique, MCF7 mammosphere cells are found to enrich breast cancer stem-like cells expressing CD44^+^CD24^-^. The stromal cells are mainly constituted by fibroblasts within a breast carcinoma, yet little is known of the contributions of the stromal cells to BCSCs.

**Methods:**

Carcinoma-associated fibroblasts (CAFs) and normal fibroblasts (NFs) were isolated and identified by immunohistochemistry. MCF7 mammosphere cells were co-cultured with different stromal fibroblasts by a transwell cocultured system. Flow cytometry was used to measure CD44 and CD24 expression status on MCF7. ELISA (enzyme-linked immunosorbent assay) was performed to investigate the production of stromal cell-derived factor 1 (SDF-1) in mammosphere cultures subject to various treatments. Mammosphere cells were injected with CAFs and NFs to examine the efficiency of tumorigenity in NOD/SCID mice.

**Results:**

CAFs derived from breast cancer patients were found to be positive for α-smooth muscle actin (α-SMA), exhibiting the traits of myofibroblasts. In addition, CAFs played a central role in promoting the proliferation of CD44^+^CD24^- ^cells through their ability to secrete SDF-1, which may be mediated to SDF-1/CXCR4 signaling. Moreover, the tumorigenicity of mammosphere cells with CAFs significantly increased as compared to that of mammosphere cells alone or with NFs.

**Conclusion:**

We for the first time investigated the effects of stromal fibroblasts on CD44^+^CD24^- ^cells and our findings indicated that breast CAFs contribute to CD44^+^CD24^- ^cell proliferation through the secretion of SDF-1, and which may be important target for therapeutic approaches.

## Introduction

Breast cancer is the most frequent malignancy among women, about 1.05 million women suffer from and 373,000 die from breast cancer per year worldwide [1]. Most recent studies indicate that breast cancer is mainly caused by breast cancer stem cells (BCSCs), and the cure for breast cancer requires BCSCs be eradicated [2,3]. In 2003, Clarke and colleagues demonstrated that a highly tumorigenic subpopulation of BCSCs, expressing CD44^+^CD24^- ^surface marker in clinical specimen, had the capacity to form tumors with as few as one hundred cells, whereas tens of thousands of the bulk breast cancer cells did not [3]. The concept of a cancer stem cell within a tumor mass, as an aberrant form of normal differentiation, is now gaining acceptance [4-6]. In order to simplify research procedure, some cancer cell lines were used to study BCSCs instead of patient samples, because they were found to have cancer stem-like cell potential. For instance, mammosphere cells were found to enrich breast cancer stem-like cells with the phenotype of CD44^+^CD24^- ^[7].

Until now, studies on breast cancer onset and development have been mainly focused on the epithelial components of the tumor, paying little attention to the surrounding tumor stromal niche. However, new evidences have emerged suggesting an important interaction between mammary epithelia and the adjacent tumor stroma. For example, only normal fibroblasts (NFs) but not carcinoma-associated fibroblasts (CAFs) exhibit the ability to inhibit the proliferation of the tumorigenic MCF10AT, suggesting that the ability of normal stromal fibroblasts to control the dysregulation of epithelial cell proliferation during breast carcinogenesis [8]. In addition, the gene expression profile of stromal fibroblasts varies widely during cancer progression, among which it includes many genes encoding secreted proteins, such as chemokines [9,10].

Chemokines are a superfamily of small molecule chemoattractive cytokines that mediate several cellular functions. SDF-1 is a member of the CXC subfamily of chemokines, and interacts with the seven-transmembrane G-protein-coupled receptor CXCR4. It is expressed by stromal cells, including fibroblasts and endothelial cells [11,12]. Normal primary mammary epithelial cells derived from different donors do not express CXCR4 mRNA [11]. In contrast, functional CXCR4 is widely expressed by different types of cancer cells. In addition, CXCR4 is found to be expressed in numerous types of embryonic and adult stem cells, which can be chemoattracted by its ligand SDF-1. Thus, it is likely that SDF-1/CXCR4 signaling plays an important role in stem cell function during the early development [13,14].

Recently, it has been reported that dysregulation in the mammary gland niche lead to abnormal expression of transforming growth factor α (TGFα), resulting in the development of breast cancer [15]. Moreover, vascular niches in brain tumors were detected to be abnormal and contributed directly to the generation of cancer stem cells and tumor growth [16]. Based on these experimental data, we hypothesized that dysregulation of the stromal niche lead to uncontrolled proliferation of stem cells, which may be the reason for tumorigenesis. In this study, we demonstrated that CAFs enhanced the expression of BCSC markers in secondary mammosphere cells and promoted the tumorigenicity of mammosphere cells in NOD/SCID mice. In addition, we proposed that SDF-1/CXCR4 signaling is involved in the cell proliferation of these cultured mammosphere cells.

## Materials and methods

### Mammosphere culture and dissociation

In our previous studies, we have showed that MCF7 cell line had the highest mammosphere-forming efficiency (MFE) among many breast cancer cells, so MCF7 cells were chosen to generate mammosphere cells in vitro [17]. Cells were then washed twice with PBS and cultured in suspension at a density of 2 × 10^5^/bottle in DMEM/F12 (HyClone, Logan, Utah) with high glucose, supplemented with 1 × B27 (Invitrogen), 20 ng/ml insulin-like growth factor I (Invitrogen), 20 ng/ml EGF (Sigma, St. Louis, MO) and 20 ng/ml b-FGF (Invitrogen). In all experiments, cells were maintained at 37°C in a humidified 5% CO_2_/95% air atmosphere. When MCF7 cells were grown in suspension for six days, "primary mammospheres" were obtained, then collected by gravity or gentle centrifugation (800 g, 10 sec), and trypsinized with 0.05% trypsin/0.53 mM EDTA-4Na (Invitrogen, Carlsbard, CA). These cells were sieved through a 40-μm nylon mesh, analyzed microscopically for single cellularity and counted. The "secondary mammospheres" were generated in culture of 2 × 10^5 ^primary mammosphere cells/bottle in the same media.

### Flow cytometry

CD24 and CD44 expression was analyzed in cells derived from monolayer cultures or in 6-day-cultured primary mammospheres following incubation in trypsin-EDTA or dissociation with a pipette and passage through a 40-μm sieve. At least 1 × 10^5 ^cells were pelleted by centrifugation at 500 g for 5 min at 4°C, resuspended in 10 μL of fluorescein isothiocyanate (FITC)-conjugated mouse anti-human CD24 monoclonal antibody and allophycocyanin (APC)-conjugated mouse anti-human CD44 monoclonal antibody (BD Pharmingen, San Diego), and incubated at 4°C in the dark for 30 to 40 min. The labeled cells were washed and then analyzed on a FACS (fluorescence activated cell sorting) Vantage (BD Biosciences).

### Quantitative real time-polymerase chain reaction (qRT-PCR)

After mammosphere cells were sorted, total RNA was extracted by using RNeasy Mini kit (Qiagen, Valencia, CA) and used for qRT-PCR assays in an ABI PRISM 7900HT sequence detection system (ABI, Norwalk, Connecticut). The specific PCR primers were used to detect the presence of *Notch2 *(F: TATTGATGACTGCCCTAA CCACA; R: ATAGCCTCCATTGCGGTTGG), *β-catenin *(F: CCTTTGTCCCGCAA ATCATG; R: ACGTACGGCGCTGGGTATC), *CXCR4 *(F: TACACCGAGGAAATG GGCTCA; R: TTCTTCACGGAAACAGGGTTC), *SDF-1 *(F: ATGCCCATGCCGA TTCTTCG; R: GCCGGGCTACAATCTGAAGG) and *GAPDH *(F: ATGGGGAAGG TGAAGGTCG; R: GGGGTCATTGATGGCAACAATA). All reactions were done in a 10-μl reaction volume in triplicate. PCR amplification consisted of 10 min of an initial denaturation step at 95°C, followed by 55 cycles of PCR at 95°C for 30 sec, 56°C for 30 sec and 72°C for 15 sec. Standard curves were generated and the relative amount of target gene mRNA was normalized to GAPDH. Specificity was verified by melt curve analysis and agarose gel electrophoresis.

### Antagonist reagents

Mammosphere cells and monolayer cells of 2 × 10^5 ^were cultured in medium (2 ml), and AMD3100, an antagonist of CXCR4, was added to the medium at 1 μg/ml. Then the cells were incubated at 37°C and 5% CO_2 _for 48 hours. qRT-PCR was used to detect *CXCR4 *expression in mammosphere cells and monolayer cells. Each experiment was conducted in triplicate.

### Tissue collection and cell preparation

Breast cancer specimens were collected from primary tumors of 4 patients who underwent surgery at Xinhua hospital. Signed informed consent was obtained from all the patients. For comparison, we have also obtained normal tissue from healthy women after plastic surgery. The tissues were minced and dissociated in DMEM/F12 supplemented with 2% bovine serum albumin, 5 mg/ml insulin, 300 U/ml collagenase and 100 U/ml hyaluronidase (all from Sigma) at 37°C for 18 h. The epithelial-cell-rich pellet was collected by centrifuging at 80 *g *for 4 min, followed by one wash with DMEM/F12. The supernatant from the first centrifugation was used as a source of mammary stromal fibroblasts. Briefly, the first supernatant were concentrated by centrifugation at 100 *g *for 10 min, and the obtained mammary stromal fibroblasts were resuspended and cultured in flasks in DMEM/F12 supplemented with 5% fetal bovine serum (Sijiqing, Hangzhou, China) and 5 mg/ml insulin. Differential trypsinization was applied during subculturing to select for the growth of fibroblasts.

### Immunohistochemistry

Coverslips with attached cells were fixed with formaldehyde for 5 min, and then stained with anti-human α-SMA (Dako, Denmark) antibody according to the manufacturer's instruction. Cells showing light brown or yellow brown grains in the cytoplasm were classified as positively staining.

### Coculture of breast stromal fibroblasts with primary mammosphere cells

Coculture of primary mammosphere cells (1 × 10^5 ^cells/dish) with breast stromal fibroblasts (1 × 10^5 ^cells/dish) were performed by using a transwell (BD) cell culture system, which allows free diffusion of substances without contact between cancer cells and stromal fibroblasts. Stromal fibroblasts in the insert layer were subcultured on a transwell cell culture membrane (7.5 cm in diameter; pore size: 0.4 μm), and mammosphere cells in the bottom layer were maintained in a 10-cm Petri dish. Stromal fibroblasts were precultured in DMEM/F12 with 10% FBS for 48 h before the start of coculture. Stromal fibroblasts were maintained in fresh serum-free DMEM/F12 medium, and mammosphere cells were cultured in suspension for six days.

### Coinoculation of mammosphere cells with different stromal fibroblasts in vivo

Mammospheres and fibroblasts were collected, enzymatically dissociated, washed in PBS, and kept at 4°C. Mice were maintained in laminar flow rooms under constant temperature and humidity and received an estradiol supplementation (0.6 mg/kg, s.i., Sigma) every 7 days for 28 days before cell injection. The mammosphere cells (1 × 10^5^) admixed with either CAFs (1 × 10^5^) or NFs (1 × 10^5^) were suspended in 0.1 ml of DMEM/F12 and then inoculated into the mammary fat pad of 5-week-old female NOD/SCID mice (Shanghai Experimental Animal Center, Chinese Academy of Sciences, Shanghai, China). Mice were examined by palpation for tumor formation for up to 12 weeks, and then were sacrificed by cervical dislocation. The histologic features of the xenografts were examined by hematoxylin and eosin staining. All experimentation performed with NOD/SCID mice, as well as routine care of the animals, was carried out in accordance with the institutional guide of animal care & use committee.

### Measurement of SDF-1

The baseline level of SDF-1 production was determined by coculture of mammosphere cells with stromal fibroblasts for six days at a density of 1 × 10^5^/bottle. The concentration of SDF-1 in the supernatant was measured by using a human SDF-1 antibody and enzyme immunoassay kit (R&D Systems, Minneapolis, MN), according to the manufacturer's instructions.

### Statistical analysis

Statistical analysis was performed by using GraphPad Prism 4.0 software© (San Diego, CA). Student's t-test (for comparison between two groups) or ANOVA with Tukey post test (for comparison between more than two groups) were used to determine whether there exists statistically significance. Fisher exact probability test was used to analyze tumorigenicity in NOD/SCID mice. Data is presented as the mean ± SEM. *P *values of ≤ 0.05 were regarded as being statistically significant.

## Results

### Primary mammosphere cells expressed higher BCSC markers and genes associated with stem cells

**Figure 1:**
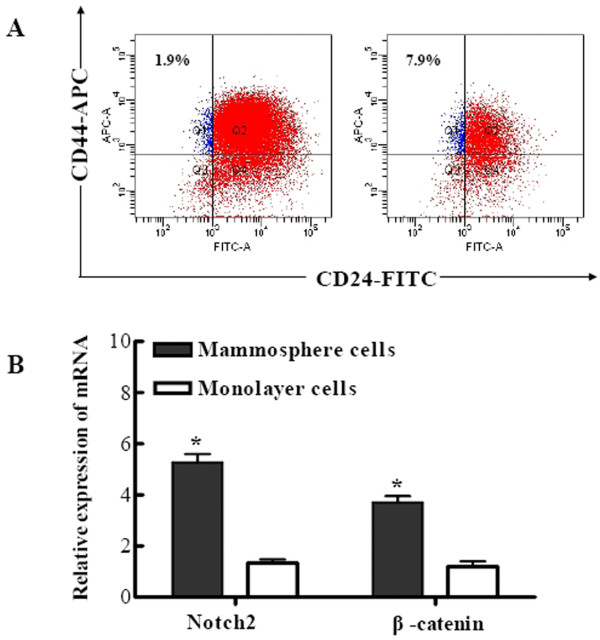
**Mammosphere cells contained subpopulations of cells expressing prospective BCSC markers**. (A) FACS analysis to measure CD44 and CD24 expression of cells derived from MCF7 monolayer cultures (left) or primary mammospheres (right), which were cultured in suspension for six days. The expression of CD44^+^CD24^- ^in mammosphere cells was (7.9 ± 0.8%), compared with (1.9 ± 0.1%) for the monolayer culture cells, *P *< 0.01. A minimum of 10,000 events were collected per sample. (B) qRT-PCR showed that Notch2 and β-catenin mRNA expression in mammosphere cells were at higher levels by around 4.0 and 3.1 fold than that in monolayer cells, respectively, *P <*0.01. The data were provided as the mean ± SD. Each experiment was performed three times.

In order to validate the generation of cancer stem-like cells through mammosphere culture, flow cytometry was used to assess the expression of BCSC marker on primary mammosphere cells and monolayer culture cells. As illustrated in Fig. 1A, when mammospheres were cultured in suspension for six days, the proportion of CD44^+^CD24^- ^cells were significantly increased as compared with that of MCF7 monolayer cells (7.9 ± 0.8% vs. 1.9 ± 0.1%, *P *< 0.01), which suggest that mammosphere cells can be used to enrich BCSCs. In addition, qRT-PCR analysis indicated that stem cell associated genes, such as *Notch2 *and *β-catenin*, were expressed in mammosphere cells at higher levels than that in monolayer cells (Fig. 1B).

### CAFs expressed high levels of α-SMA

**Figure 2:**
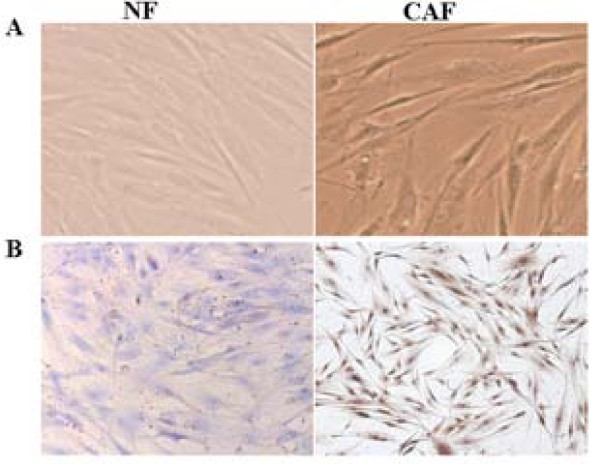
**Immunohistochemistry of NFs and CAFs**. (A) Phase images of primary cultures of stromal fibroblasts isolated from invasive ductal carcinomas (right) and stromal fibroblasts from normal breast tissue (left), original magnification × 100. (B) CAFs (right) were positive for *α*-SMA staining, while NFs (left) were negative.

Primary stromal fibroblasts were cultured in DMEM/F12 supplemented with 5% fetal bovine serum and 5 mg/ml insulin, and no epithelial cells were detected in passage 3 stromal fibroblasts. Although the morphology and growth pattern of CAFs and NFs was similar (Fig. 2A), immunohistochemical staining showed that CAFs exhibited strongly positive expression of α-SMA, whereas NFs did not (Fig. 2B). In addition, this increased expression of α-SMA in CAFs was maintained for up to eight passages in vitro, indicating that isolated CAFs contained a high proportion of myofibroblasts.

### CAFs promoted the generation of CD44^+^CD24^- ^cells in mammosphere cells

**Figure 3:**
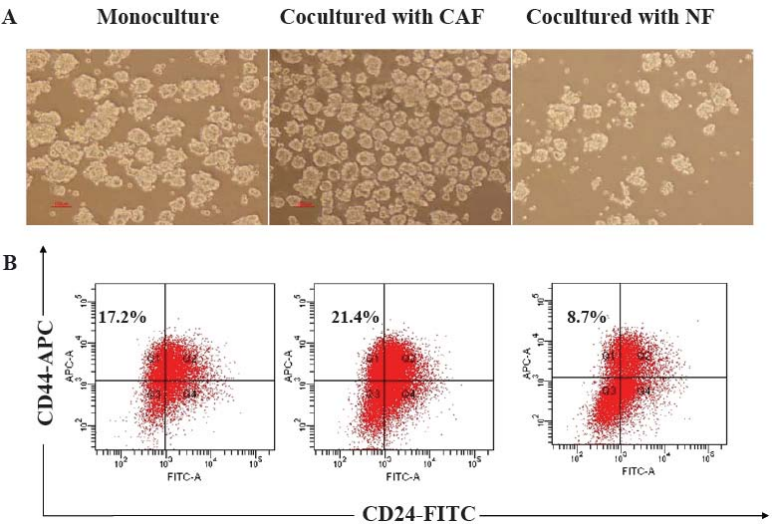
**Mammosphere cells were cocultured with different stromal fibroblasts and flow cytometry was used to measure CD44 and CD24 expression**. (A) Mammosphere cells (1 × 10^5 ^cells/dish) cocultured with different stromal fibroblasts (1 × 10^5 ^cells/dish) using transwells for six days, and mammosphere cells cocultured with CAFs (middle) had the highest MFE (13.5 ± 1.2%), compared with monoculture mammosphere cells (left) (8.1 ± 0.7%), *P *< 0.01. (B) Flow cytometry analysis to measure CD44 and CD24 expression of cells derived from monoculture mammosphere cells and cocultured mammosphere cells. The expression of CD44^+^CD24^- ^in monoculture mammosphere cells (left) was (17.2 ± 2.3%). Compared to monoculture mammosphere cells, the expression of CD44^+^CD24^- ^in cocultured mammosphere cells with CAFs (middle) was (21.4 ± 1.8%), *P *< 0.05, and the expression of CD44^+^CD24^- ^in cocultured mammosphere cells with NFs (right) was (8.7 ± 0.9%), *P *< 0.01. The data were provided as the mean ± SD. Each experiment was performed three times.

To determine whether CAFs affect the generation of cancer stem-like cells in mammosphere cells, we cocultured primary mammosphere cells with stromal fibroblasts in transwells for six days. It was observed that cocultured mammosphere cells with CAFs siginicantly increased MFE (13.5 ± 1.2% vs. 8.1 ± 0.7, *P *< 0.01), and mammosphere cell number (3.82 ± 0.41 × 10^5 ^vs. 1.51 ± 0.43, *P *< 0.01) as compared to that of mammosphere cells culture alone. In contrast, NFs markedly inhibit MFE (5.2 ± 0.6 % vs. 8.1 ± 0.7, *P *< 0.05), and cell number (0.65 ± 0.22 × 10^5 ^vs. 1.51 ± 0.43, *P *< 0.01) as compared to that of mammosphere cells culture alone (Table 1 and Fig. 3A). In addition, we used flow cytometry to assess the proportion of BCSCs that has the phenotypic marker of CD44^+^CD24^-^, and found that CAFs significantly increased the proportion of CD44^+^CD24^- ^cells in mammospheres (21.4 ± 1.8% vs. 17.2 ± 2.3%, *P *< 0.05); while NFs decreased the proportion of CD44^+^CD24^- ^cells in mammospheres (8.7 ± 0.9% vs. 17.2 ± 2.3%, *P *< 0.01) (Fig. 3B, and see Additional file 1), which exhibited similar trend as MFE. These results suggest that CAFs have positive effects on the generation of CD44^+^CD24^- ^cells, while NFs have negative effects on CD44^+^CD24^- ^cell formation.

**Table 1:** Different MFE and cell number when cocultured with different stromal fibroblasts

Culture Condition	MFE (%)	**Cell Number (× 10**^**5**^)
Monoculture	8.1 ± 0.7	1.51 ± 0.43
Mammosphere + CAFs	13.5 ± 1.2**	3.82 ± 0.41**
Mammosphere + NFs	5.2 ± 0.6*	0.65 ± 0.22*

*P < 0.05, **P < 0.01 compared with monoculture

### CAFs had a positive role on the tumorigenicity of mammosphere cells

To investigate whether altered stromal niche could influence the tumorigenicity in vivo, we evaluated the tumor formation in NOD/SCID mice by inoculation of mammosphere cells with or without CAFs and NFs. The results revealed that inoculation of 1 × 10^5 ^mammosphere cells alone resulted in tumor formation in 60% of mice (3/5), and coinoculation of 1 × 10^5 ^mammosphere cells with 1 × 10^5 ^CAFs significantly improved tumor formation (5/5). Interestingly, coinoculation of 1 × 10^5 ^mammosphere cells with 1 × 10^5 ^NFs sharply decreased tumorigenicity, only 20% mice developed tumors (1/5, Table 2). These data strongly suggested that cancer stromal fibroblast significantly promote the tumorigenicity of mammosphere cells.

**Table 2:** Incidence of tumors by coinoculation of mammosphere cells with CAFs and NFs in NOD/SCID mice

Cells Inoculated	Mammosphere	Mammosphere + CAFs	Mammosphere + NFs
Tumors	3/5	5/5*	1/5*

*P < 0.01 compared with the same inoculation number of mammosphere cells alone.

### The cocultured medium of primary mammosphere cells with CAFs had higher SDF-1 expression

**Figure 4:**
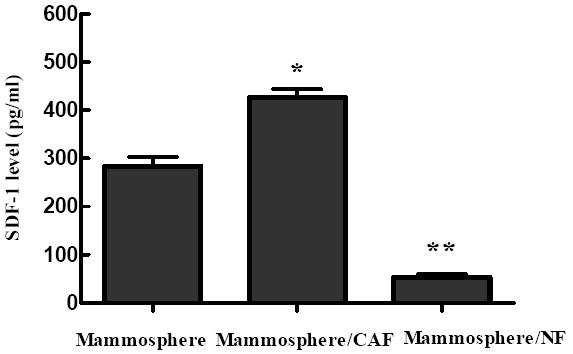
**The SDF-1 protein expression in cocultured medium of mammosphere cells with CAFs and NFs**. The SDF-1 protein level in the medium conditioned by the CAFs was (426.4 ± 30.6) (pg/ml) (middle), compared to the levels produced by mammosphere cells alone (283.6 ± 35.1) (pg/ml) (left), *P <*0.05. The cocultured medium of mammosphere cells with NFs (right) showed a far lower level of SDF-1(52.9. ± 13.1) (pg/ml) secretion when compared with mammosphere cells alone, *P <*0.01. The SDF-1 level was measured three times in each experiment.

The marked effects of cancer stromal niche promote us to investigate the molecular mechanisms by which CAFs increased the tumorigenicity of mammosphere cells. Recent reports have indicated that SDF-1 boosts the proliferation of several cancer cell lines in culture, including breast carcinoma cells [10]. In order to determine whether SDF-1 involved in the proliferation of CD44^+^CD24^- ^cells, the production of SDF-1 in mammosphere cultures subject to various treatments were measured by ELISA. The result indicated elevated levels of SDF-1 protein in the medium conditioned by the CAFs as compared with that by mammosphere cells alone (426.4 ± 30.6 pg/ml vs. 283.6 ± 35.1 pg/ml, *P *< 0.05). In addition, the cocultured medium of mammosphere cells with NFs significantly decreased the production of SDF-1 (52.9. ± 13.1 pg/ml vs. 283.6 ± 35.1 pg/ml, *P <*0.01) (Fig. 4). These results exhibited the similar trend as MFE, generation of CD44^+^CD24^- ^cells and tumorigenicity of mammosphere cells by CAFs, implying that the elevated production of SDF-1 by CAFs may be the reason for the promoted MFE, generation of CD44^+^CD24^- ^cells and tumorigenicity of mammosphere cells.

### CXCR4 antagonist reduced the generation of CD44^+^CD24^- ^cells

**Figure 5:**
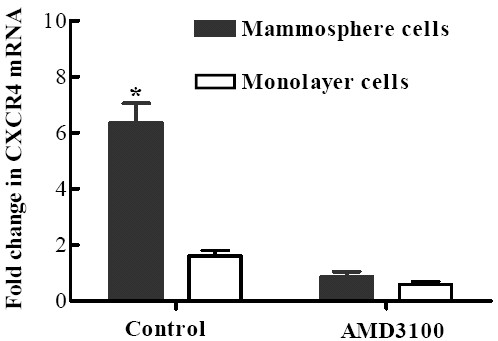
**Mammosphere cells and monolayer cells were cultured in the presence of 1 μg/ml AMD3100 for 48 h**. qRT-PCR showed that CXCR4 mRNA expression in mammosphere cells was 3.9 fold higher than that in monolayer cells, (*P <*0.01), and AMD3100 could significantly down-regulate it in both when treated for 48 h.

**Figure 6:**
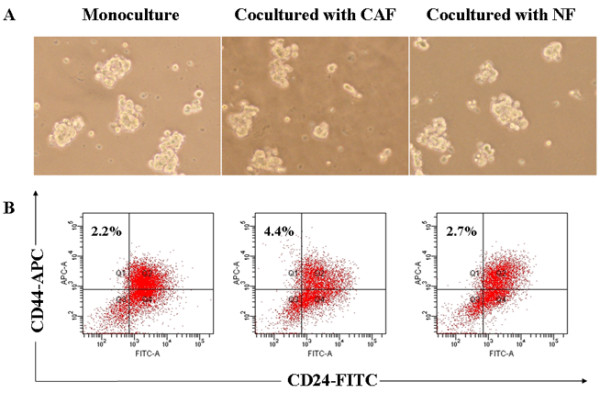
**Mammosphere cells were cocultured with different stromal fibroblasts with the administration of AMD3100 and flow cytometry was used to measure CD44 and CD24 expression**. (A) Mammosphere cells were cocultured with different stromal fibroblasts with the administration of AMD3100 (1 μg/ml) for six days. As a result, MFE in monoculture mammosphere cells (left), cocultured mammosphere cells with CAFs (middle) and NFs (right) was significantly reduced to (1.6 ± 0.1%), (2.3 ± 0.2%) and (1.5 ± 0.2%), respectively. (B) Flow cytometry analysis was used to measure CD44 and CD24 expression of cells derived from mammosphere cells. The expression of CD44^+^CD24^- ^in monoculture mammosphere cells (left), cocultured mammosphere cells with stromal CAFs (middle) and NFs (right) was (2.2 ± 0.3%), (4.4 ± 0.8%) and (2.7 ± 0.3%), respectively. The data were provided as the mean ± SD. Each experiment was performed three times.

In order to further prove whether enhanced generation of CD44^+^CD24^- ^cells by CAFs is mediated by SDF-1 and its receptor CXCR4, we detected CXCR4 expression in mammosphere cells and monolayer cells by qRT-PCR. The results showed that CXCR4 mRNA expression was higher in mammosphere cells than that in monolayer cells, (*P *< 0.01, Fig. 5), and CXCR4 antagonist AMD3100 could decrease CXCR4 gene expression in both cells. Moreover, AMD3100 significantly reduced MFE and mammosphere cell number in monoculture mammospheres and cocultured mammospheres with CAFs and NFs (Table 3), and decreased the proportion of CD44^+^CD24^- ^cells (Fig. 6, and see Additional file 2). These results collectively demonstrated that CAFs enhanced generation of CD44^+^CD24^- ^cells in mammospheres may be caused by SDF-1/CXCR4 signaling.

**Table 3:** AMD3100 significantly inhibited MFE and cell number when cocultured with different stromal fibroblasts

Culture Condition	MFE (%)	**Cell Number (× 10**^**5**^)
Monoculture	1.6 ± 0.1	0.22 ± 0.07
Mammosphere + CAFs	2.3 ± 0.2	0.43 ± 0.14
Mammosphere + NFs	1.5 ± 0.2	0.28 ± 0.08

*P < 0.01 compared with no treatment of AMD3100.

## Discussion

Mammosphere culture system is now widely used for stem cell culture. Dontu and his colleagues had developed an in vitro cultivation system that allowed for the proliferation of undifferentiated human mammary epithelial cells in suspension. When cultured on nonadherent surfaces in the presence of growth factors, nonadherent mammospheres were enriched in cells with functional characteristics of stem/progenitor cells [18]. Another study also showed that breast tumorigenic cells with self-renewal could be propagated in vitro as nonadherent mammospheres [7]. Consistent with the above reports, our study shows that mammosphere cells could be cultured in suspension and generate BCSCs with the CD44^+^CD24^- ^phenotype. Thus, long-term cultures of mammosphere in vitro may represent a suitable model to study BCSCs.

Stem cell properties in normal and malignant tissues are tightly regulated by the Wnt, Shh and Notch signaling pathways [19-21]. Notch signaling has been implicated in the regulation of cell-fate decisions such as self-renewal of adult stem cells and differentiation of progenitor cells along a particular lineage. Dontu and his colleagues demonstrated that Notch activation promoted mammary stem cell self-renewal, but modulation of this pathway had no significant effect on differentiated mammary epithelial cells [20]. In breast cancers, it was found that BCSCs preferentially expressed some "stemness" genes, including *Notch1 *and *β-catenin *[18]. Our qRT-PCR analysis obtained the similar result that *Notch2 *and *β-catenin *were expressed at higher levels in mammosphere cells than in monolayer cells, suggesting that *Notch2 *and *β-catenin *are involved in BCSC regulation.

Recent studies have indicated that tumor niches play an important role in regulating the growth and metastasis of primary tumors. For example, in cocultured experiments, CAFs extracted from human breast carcinomas were more competent in promoting the growth of admixed breast carcinoma cells than NFs that derived from the same patients [22]. Similarly, when exposed to the conditioned medium of pancreatic stellate cells isolated from resected pancreatic adenocarcinoma, pancreatic epithelial cells showed an increase in proliferation, migration, invasion and colony formation in soft agar in a dose-dependent manner [2,3].

It is well known that expression of α-SMA is a defining characteristic of myofibroblasts [24], which activates the growth of fibroblasts in areas of inflammation during wound healing [25]. Our results demonstrated that human mammary carcinomas, from which we had extracted CAFs, carried large numbers of myofibroblasts in their stroma. In this study, we found that CAFs up-regulated the proportion of CD44^+^CD24^- ^cells in mammospheres, whereas NFs down-regulated it in mammospheres, implying that the CAFs have positive effects on CD44^+^CD24^- ^cell generation, while NFs have negative effects on it. Furthermore, coinoculation of mammosphere cells with CAFs into NOD/SCID mice significantly increased tumorigenicity as compared to those obtained with mammosphere cells alone or with NFs. This might be attributed to the enhanced generation of mammosphere CD44^+^CD24^- ^cells by CAFs.

Importantly, endogenous CXCR4 expression on carcinoma cells is known to correlate with a poor prognosis for several types of carcinomas [26,27]. The knockdown of CXCR4 expression by a small interfering RNA in breast carcinoma cells decreases cell invasion and proliferation in vitro and abrogates the tumor growth in vivo [28,29]. Furthermore, the selective blocking of the CXCR4 by plerixafor overcome the protective effect of the bone marrow environment for BCR-ABL(+) leukemia [30]. Consistent with the above findings, our results suggested that *CXCR4 *gene is expressed in mammosphere cells at higher levels than that in monolayer cells. So we hypothesized that CAFs enhanced the proliferation of CD44^+^CD24^- ^cells in secondary mammosphere cells through CXCR4.

Essential SDF-1/CXCR4 interactions have been increasingly demonstrated in various tissues and culture systems and it is possible that SDF-1/CXCR4 initiated different signal pathways for cell proliferation and migration [27,31,32]. In malignant tumors, SDF-1/CXCR4 may provide paracrine signals in promoting malignant progression such as metastasis, invasion and cell proliferation [33-35]. We found in this study that SDF-1 was highly released in the conditioned medium of mammosphere cells with CAFs, compared with NFs. In addition, the interaction of SDF-1 released from CAFs and CRCX4 expressed on mammosphere cells is at least partly involved in the proliferation of mammosphere. Thus, it is likely that SDF-1 secreted by stromal myofibroblasts significantly affects CXCR4-expressing mammosphere cells through direct paracrine stimulation. This was further proved by CXCR4 antagonist AMD3100, which significantly reduced MFE and the expression of BCSC markers in secondary mammosphere cells.

Collectively, these data indicated that the specific interactions of SDF-1 with their receptor CXCR4 that expressed on mammosphere cells are likely to occur in tumor-stromal niches, and these interactions may be responsible for the proliferation of CD44^+^CD24^- ^cells. The proliferation of mammosphere cells was observed to be promoted by being cocultured with CAFs, suggesting that SDF-1/CXCR4 signaling is involved in the cell proliferation of these cocultured mammosphere cells. CXCR4 and SDF-1 are candidate factors that involved in the cross-talk of the tumor-niche interaction of CD44^+^CD24^- ^cells. Because the increase in the proliferation of cocultured mammosphere cells induced by SDF-1 was completely inhibited by AMD3100, therapeutic strategies that target SDF-1/CXCR4 may be beneficial to breast cancer patients. So, new strategies need to take into account the role of the niches that can have a critical role in modulating BCSCs and response to therapeutic agents. It should be noted that this study had only examined the interaction of stromal fibroblasts and CD44^+^CD24^- ^cells in two dimensions, and how they interact with each other in three-dimensional culture remains to be further studied.

## Competing interests

The authors declare that they have no competing interests.

## Authors' contributions

MZH conceived of the study, carried out the experimental studies, and drafted the manuscript. YQL participated in the design of the study and performed the data analysis. HLZ and FFN participated in its design and helped to draft the manuscript. All authors read and approved the final manuscript.

## Supplementary Material

Additional file 1 **Additional samples analyzed with FACS as described in legend for Figure **3. The data provided represent the other two tests analyzed with FACS as described in legend for Figure 3. Click here for file

Additional file 2 **Additional samples analyzed with FACS as described in legend for Figure **6. The data provided represent the other two tests analyzed with FACS as described in legend for Figure 6. Click here for file
