# Supplementary figures and images for: Breast cancer stromal fibroblasts promote the generation of CD44+CD24- cells through SDF-1/CXCR4 interaction
Source: J Exp Clin Cancer Res. 2010 Jun 22;29(1):80. doi: 10.1186/1756-9966-29-80 (PMC2911413; doi:10.1186/1756-9966-29-80)

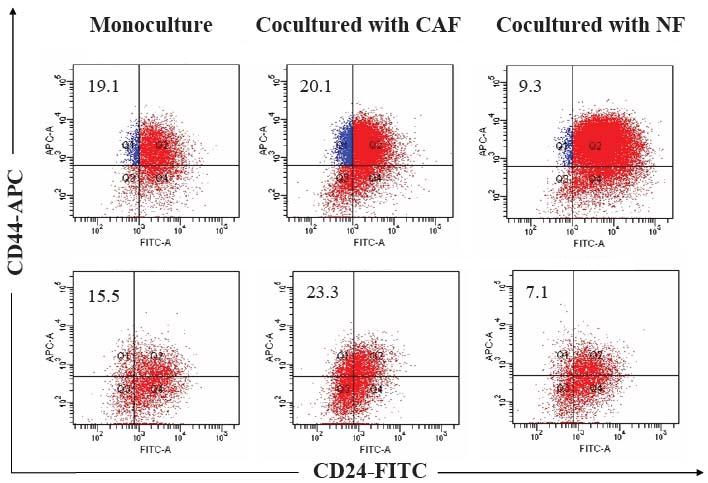

Supplement: Additional file 1 — Additional samples analyzed with FACS as described in legend for Figure 3. The data provided represent the other two tests analyzed with FACS as described in legend for Figure 3. [file 1756-9966-29-80-S1.JPEG]

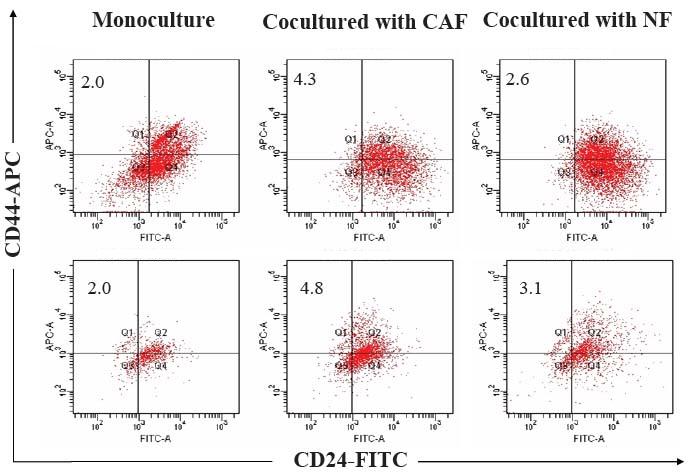

Supplement: Additional file 2 — Additional samples analyzed with FACS as described in legend for Figure 6. The data provided represent the other two tests analyzed with FACS as described in legend for Figure 6. [file 1756-9966-29-80-S2.JPEG]
